# Identification method and application of order parameters for organizational systems considering time-series values: A case study on high-quality development in China’s regional manufacturing industry

**DOI:** 10.1371/journal.pone.0319267

**Published:** 2025-03-31

**Authors:** Xin Wen, Yuqing Zhou

**Affiliations:** School of Management, Shenyang University of Technology, Liaoning, China; Ataturk University, TÜRKIYE

## Abstract

The transition from disorder to order in the evolution of organizational systems is driven by the identification of key order parameters, which serve as critical determinants of this shift and the primary forces behind system evolution. Since order parameters capture the dominant state of the system at specific time points, accurately identifying these parameters is essential for tracing the evolutionary trajectory. This study integrates time-series values into the process of order parameter identification and employs the CRITIC method to objectively calculate the weights of these values, thereby enhancing the scientific rigor and effectiveness of the identification process. The results indicate that the identified order parameters accurately reflect the actual evolutionary state of the system. Furthermore, this method efficiently controls order parameters throughout the evolution of the system, providing operational and technical references for subsequent development processes. By focusing on a case study of high-quality development in the manufacturing industry in China, this study validates the practical application of the proposed method and highlights the significance of order parameter identification in enhancing organizational system development.

## Introduction

In recent years, digitalization has become the dominant direction of social and organizational change. As digitalization becomes an irreversible trend, Chinese enterprises are actively exploring its potential [1]. According to the Digital Change Index of Chinese Enterprises 2023, over half (53%) of Chinese companies plan to further increase their investment in digitalization. However, there remains a significant disparity between Chinese social organizations and those in developed countries. This suggests that while current models for social organization and enterprise development have facilitated rapid growth, they are insufficient for adapting to the rapidly changing market and trends of the times. On one hand, social organizations have not paid sufficient attention to their previous basic achievements in the development process and have not fully mobilized their resources to make full use of their comparative advantages. On the other hand, historical decisions are a significant factor in an organization's development, and their value plays a crucial role in current decision-making. The self-worth of the organization is essential [2]. To meet the challenge of mastering its own developmental patterns and strengths, the organization must focus on them. Multiple order parameters exist in the process of organizational development, and their effects vary over time. These parameters play a key role in the organization's current decision-making [3].

Accurate identification of order parameters is crucial for understanding the evolution of organizational systems and making informed decisions. Order parameters can reflect the dominant state and evolution trends of a system at specific time points, providing key insights for decision-making. In the context of digital transformation, the development of organizational systems becomes more complex and variable, increasing the difficulty of identifying order parameters. There is an urgent need for a new method of order parameter identification that can fully consider the characteristics of time-series data and accurately capture the state changes of organizational systems at different time points, providing strong support for organizational development. Therefore, this study proposes a novel order parameter identification model that integrates time-series analysis with the *CRITIC* method. By incorporating time-series data, the model can more accurately capture the state changes of organizational systems at different time points, revealing the dynamic patterns of system evolution. Compared to traditional methods, this model demonstrates greater adaptability and accuracy in handling time-series data, better reflecting the true evolution process of the system. Starting from the perspective of order parameters, this study offers new insights into the high-quality development of the manufacturing industry. By identifying key order parameters, it clarifies the critical influencing factors and driving forces behind high-quality development in manufacturing, providing a scientific basis for governments and enterprises to formulate relevant policies and strategies. Additionally, the study proposes corresponding regulatory strategies, which have significant practical guidance value.

The core question of this study is: How can order parameters in organizational systems be accurately identified when considering time-series values, and what are the application effects and influencing factors of these parameters in the high-quality development of the manufacturing industry? Specifically, the following questions need to be addressed: How does time-series data affect the identification of order parameters? What are the characteristics and patterns of order parameters at different time points? What are the mechanisms by which order parameters influence the high-quality development of the manufacturing industry? How can effective regulatory strategies be formulated based on the identification results of order parameters? The study is structured as follows: Section 2 provides a literature review, summarizing existing literature. Section 3 introduces the order parameter identification model. Section 4 describes the regulatory strategies. Section 5 presents a practical example of the model. Section 6 delves into the significance of incorporating time-series values into the identification of order parameters within organizational systems, with a particular focus on regional manufacturing in China, and offers conclusions and recommendations for future research.

## Literature review

In this section, the literature review is presented on models used for identifying order parameters, their applications across various contexts, and their significance in synergistic system development. On this basis, the existing literature is summarized, and the research objectives of this paper are proposed.

### Models for identifying order parameter

Order parameter identification models have been widely applied across various fields to optimize and enhance system performance by capturing key variables that influence system behavior. Different models employ advanced techniques such as evolutionary algorithms and decomposition methods to accurately identify these parameters. Zheng et al. [4] conducted a comprehensive evaluation of multiple ordinal covariates in the system using three computational methods. Park et al. [5] propose a dynamic re-order parameter to reveal subtle phase transitions in interacting magnets, aligning with the synergetic role of order parameters in uncovering hidden material phases. Sun et al. [6] developed a model based on the coordination degree of system evolution rate and trend, aiming to reflect dynamic changes in the level of industrial development. The order parameter identification method based on output/input perspective analysis was found to effectively promote the efficiency of the system evolution process [7]. In addition, Stark et al. [8] proposed a method for parameter and order identification in fractional order systems, particularly suitable for lithium-ion batteries. Kos et al. [9] introduced a simple analytical method for estimating a five-parameter model with zero-addition time delay. Gu et al. [10] introduced the L-SHADED method to identify photovoltaic model parameters under varying temperature and irradiance conditions, significantly improving identification accuracy. Hossain et al. [11] developed a multifunctional material model based on the Preisach hysteresis operator, achieving thermodynamically consistent parameter identification. These studies highlight the importance of order parameter identification in complex systems, as accurate identification is essential for improving model precision and applicability, whether in energy systems or multifunctional materials.

### Practical applications of order parameters

The theory of order parameters, as a critical tool for describing the states and evolution of complex systems, has been widely applied across various disciplines. Its applications span from time-series analysis to material science. Santoro et al. [12] revealed complex dynamic patterns in multivariate time series through a higher-order dependency framework, with applications in both neuroscience and economics. Liu et al. [13] reviewed the role of machine learning in phase transitions and the modeling of material properties in high-entropy alloys, demonstrating the importance of order parameters in the design of complex materials. Malizia et al. [14] proposed a method for reconstructing higher-order interactions in coupled systems, showcasing its application in microscopic dynamical systems. Wen et al. [15] used order parameters combined with the TOPSIS method to assess the digital transformation capabilities of the manufacturing industry, revealing the influence of regional economic development on transformation capacity. The wide application of order parameters provides theoretical support for analyzing various complex systems, driving innovation and development in fields ranging from dynamic time-series patterns to material design.

### The role of order parameter in the synergistic development of systems

In the study of system synergetic development, order parameters play a crucial dominant role as key elements of system evolution. Numerous empirical studies across different fields have explored the role of order parameters in coordination mechanisms. Hu et al. [16] demonstrated how energy intensity, as an order parameter, drives the system toward a higher level of synergy. Du and Wang [17] showed that the order parameter shifted from manufacturing to logistics in the coevolution of the two industries, aligning with synergetic principles. Fang et al. [18] focused on the integration of rural cultural tourism public services, using the Haken model to analyze the role of service function integration as an order parameter, pointing out that it is a necessary stage for synergetic development and plays a critical dominant role in regional integration. Alikhani et al. [19] highlight how order parameter coordination, through the synergistic interaction of resilience strategies, plays a crucial role in optimizing supply chain networks under disruption. Bai et al. [20] show that synergistic allocation acts as an order parameter, driving technological innovation through coordinated organizational slack. Collectively, these studies underscore the pivotal role of order parameters in system coordination across various fields, illustrating that identifying key order parameters can guide systems toward more efficient synergetic states.

### Literature summary

Based on the literature review, it is evident that the existing studies mainly focus on the macro-level interactions of order parameters or the impact of system states on the evolutionary processes of organizations. However, limited attention has been given to the dynamics and patterns of organizational system development at different stages of evolution. This paper addresses this gap by utilizing the *CRITIC* method to calculate the value weights of cross-sectional data at each time point within the organizational development time series, based on the identification of order parameters. Furthermore, this study proposes an order parameter identification model that incorporates time-series values, offering a more comprehensive approach to understanding how order parameters are identified and applied in organizational systems, particularly in the context of considering time-series values.

### The identification method of the order parameter of the organization system considering time-series value

In the process of organizational system evolution, the transition from disorder to order is driven by the identification of key order parameters. These parameters are not only critical determinants of the shift towards order but also the primary forces behind its evolution [21]. Since order parameters capture the dominant state of the system at specific points in time, accurately identifying these parameters is essential for tracing the evolutionary trajectory of the system. To effectively capture the state of the system at each time point, this study integrates time-series values into the process of order parameter identification and employs the *CRITIC* method to calculate the weights of each time point. The *CRITIC* method objectively assigns appropriate weights based on the variation in cross-sectional data across different time points, ensuring that the identified order parameters accurately reflect the actual evolutionary state of the system.

The *CRITIC* method was selected over other weighting methods for the following reasons:

[1] Objectivity and Applicability: the *CRITIC* method is an objective weighting approach that relies on the inherent characteristics of evaluation criteria. It assesses the importance of each criterion by evaluating the contrast intensity and conflict between them. This objectivity minimizes the influence of subjective factors, such as expert scoring, which is a common limitation in many other weighting methods. In this study, the *CRITIC* method is particularly suitable for handling time-series data in organizational systems, ensuring that the weights assigned to different order parameters are based on data-driven insights rather than human judgment.
[2] Compatibility with Time-Series Data: The *CRITIC* method effectively captures differences between time points by evaluating the relative importance of time-series data, with particular attention to conflicts and contrasts between indicators. Given that the state of organizational systems evolves over time, researchers can use *CRITIC* method to weigh these changes, ensuring accurate identification of dominant order parameters at different time points. This makes the method highly compatible with the time-series analysis in this study, allowing it to reflect the dynamic characteristics of the system’ss evolution.
[3] Comparison with Alternative Methods: Researchers considered several alternative weighting methods, including the entropy method and AHP (Analytic Hierarchy Process). However, the entropy method often fails to account for conflicts between indicators, dominant to potential biases in complex systems. AHP, while powerful in structured decision-making, introduces subjectivity by relying on expert judgment. In contrast, *CRITIC* method not only considers interdependencies between indicators but also maintains objectivity and consistency in weight allocation, making it more adaptable and scientifically robust for analyzing the evolution of organizational systems.
[4] Relevance to Research Question: This study aims to analyze the state changes of organizational systems over time through time-series data. *CRITIC* method can assign reasonable weights to order parameters at different time points based on cross-sectional data. This ensures that the identified order parameters at each time point align with the actual state of the system, which is crucial for addressing the research question of how to capture the dynamic evolution of the system using time-series data. Therefore, the integration of *CRITIC* method strengthens the scientific foundation of the study and ensures that the model accurately captures the evolution of the system.

### The order parameter identification model at the time point in the system development

By using the cross-sectional data of each time point in the process of organizational system development, this paper constructs the evaluation index system of the organizational system and probes into the dominant order parameter at each time point in the process of organizational system evolution and development. According to the development level of the organization under the index system, the structure of the system’s personality value parameters is calculated.

The determination of the parameter structure of personality value:


$$d(x_{ti},x_{t}{}^{*})=\sqrt{\sum_{j=1}^{p}w_{tij}^{2}}{\left(x_{tj}^{*}-x_{ti}\right)}^{2}$$ (1)

*t* stands for any time point in the past; $x_{t}*$ in the formula is the ideal result of the system under the evaluation index at Time Point *t*; $x_{ti}={\left(x_{ti1},x_{ti2},...x_{tip}\right)}^{\tau }$ is the index value vector of subsystem *i* normalized at the time point *t*; $w_{ti}={\left(w_{ti1},w_{ti2},...w_{tip}\right)}^{\tau }$ represents the parameter structure of personality value of subsystem *i* at the time point *t*.

In this paper, we assume that the subsystem *i*, from its most advantageous point of view, determines the value parameter structure of personality at the time point *t*; based on the idea of goal planning, this paper constructs a mathematical model to identify the main melody of each subsystem at each time point *t* in the development process of an organization system. This assumption is based on the premise that each subsystem operates under relatively stable conditions and is influenced by its internal capabilities and external environment. This allows for the identification of the dominant order parameter based on the system’s specific conditions at each time point. However, this assumption may not hold if the external environment undergoes significant changes, which could affect the capacity to maintain its most advantageous position. In such cases, additional adjustment mechanisms would be required to account for dynamic fluctuations in the external environment.


$$\min d_{ti}^{2}(x_{ti},x_{t}{}^{*})=\overset{p}{\underset{j=1}{\sum }}w_{tij}^{2}{\left(x_{tj}^{*}-x_{ti}\right)}^{2}$$ (2)

$$s.t.\sum_{j=1}^{p}w_{tij}=1$$

$$w_{tij}\geq 0$$

$$t=(1,2,...,\partial );j=\left(1,2,...,p\right);i\text{=}\left(\text{1,2,}...\text{,n}\right)$$

$w_{ti}$ is the parameter structure of the personality value of the subsystem *i* at the time point *t* and is the dominant structure of the subsystem *i* in the evaluation system. Moreover, this mathematical model of the parameter structure of personality is as follows:


$$\begin{array}{l} w_{tij}^{\text{*}}=\frac{{\text{λ}}_{t}}{{\left({\text{x}}_{\text{ti}}^{\text{*}}-{\text{x}}_{\text{tij}}\right)}^{2}}\text{i}=1,2,\ldots ,\text{n}\\ {\text{λ}}_{t}\text{*}=\frac{1}{{\sum^{\text{​}}}_{\text{j}=1}^{\text{p}}\frac{1}{{\left({\text{x}}_{\text{ti}}^{\text{*}}-{\text{x}}_{\text{tij}}\right)}^{2}}} \end{array}$$ (3)

The value parameter structure of the subsystem *i* at the time point *t* is as follows:


$$w_{ti}={\left(w_{ti1},w_{ti2},\ldots ,w_{tip}\right)}^{\tau }$$

Cluster analysis of Set ${\text{w}}_{t}=\left\{{\text{w}}_{tij}|i=1,2,\ldots ,\text{n},j=1,2,\ldots ,p;t=1,2,...,\partial \right\}$, which is made up of the value parameter structure of each subsystem at the time point t, can obtain the main melody of the groups to which each subsystem belongs.

System ideal value parameter structure


$${\text{w}}_{tj}{}^{\text{*}}=\frac{1}{\text{n}}\sum_{i=1}^{n}w_{tij}$$ (4)

The value parameter structure of the main melody of each group at the time point *t* is introduced into the objective benefit function, and the order of all $\text{d}\left({\text{x}}_{\text{ti}},{\text{x}}_{t}*\right)$ is the ideal order structure. In the same way, we can calculate the different order structures of each main melody at the time point *t*. According to the similarity between the order structure $I_{tk}^{\text{*}}$ and the ideal order structure $I_{tr}^{\text{*}}$ in the main melody, the dominant order parameter can be determined at the time point *t*.

When the time point *t*, the subsystem *i* is in the ideal value parameter structure situation of the benefit evaluation value.


$$\text{d}\left(x_{ti},x_{t}^{*}\right)=\sqrt{\sum_{j=1}^{p}w_{tij}^{2}}{\left(x_{tj}^{*}-x_{tij}\right)}^{2}=\sqrt{\sum_{j=1}^{p}{\left(\frac{1}{n}\sum_{i=1}^{n}w_{ti}^{*}\right)}^{2}{\left(x_{tj}^{*}-x_{tij}\right)}^{2}}$$ (5)

The deviation between order structure one and the ideal order structure two


$$d_{trk}=\sqrt{\overset{n}{\underset{i=1}{\sum }}{\left(I_{tri}^{\text{*}}-I_{tki}^{\text{*}}\right)}^{2}}$$ (6)

Expressed in terms of similarity coefficient:


$$C_{tRk}=\frac{1}{1+d_{trk}}$$ (7)

The main melody with the most significant similarity coefficient has the highest similarity degree if the distance between it and the ideal value parameter is the smallest. The value parameter structure of the main melody $C_{tRk}$ is the dominant order parameter $\lambda_{ti}$ of the transformation and development of the system at the time point *t*.

### Based on the *CRITIC* weight method, the time-series value of cross-section data of the time point in the development of the organization system is calculated

#### Time-value evolution benefit function of time-point cross-section data.

In economics, the value of time represents a concrete and objective measure of labor productivity. However, in practice, the time-series value is influenced by numerous uncertain factors, making accurate calculation challenging. Drawing from the theory of consumer behavior and the principle of optimization, human activities—such as production and management—are modeled through a benefit function that aims to maximize overall benefit by selecting an optimal combination of activities [22]. According to this principle, each individual index provides only a partial reflection of the organizational system’s state when determining the time-series value of cross-sectional data at different time points. A comprehensive and accurate evaluation of an organization’s development necessitates the integration of all relevant indices [23]. Therefore, constructing a time-value evolution benefit function based on past time-point cross-sectional data is essential for aligning the evaluation with actual outcomes.

This assumption is grounded in the inherent complexity of real-world organizational development, where external factors such as market fluctuations, technological advancements, and policy changes significantly influence evolution of the system. The application of the benefit function derived from consumer behavior theory effectively captures the dynamic allocation of resources within an evolving system. However, this assumption has limitations, particularly its dependence on relatively stable market conditions. In the event of sudden and extreme changes, such as financial crises or significant policy shifts, the model may require recalibration or the incorporation of scenario-based analyses to maintain predictive accuracy.

The basic assumptions are as follows:


$$Z(x_{ti})=\sum_{j=1}^{p}w_{tij}\frac{f(x_{tij})}{x_{tij}}$$ (8)

The symbol $Z(x_{ti})$ denotes the evolutionary value benefit of the time-series value of the cross-sectional data of the organization system at the past time point *t*. In this paper, the subsystem *i* is selected as the object of evaluation. It is assumed that the evaluation system of the development level of the system includes *p* evaluation indexes, and these indexes are taken as the evaluation samples. It is considered that the time series contains  ∂  time points, and the numbers of these time points are $\left\{t_{1},t_{2},...,t_{\partial }\right\}$. $f(x_{tij})$ indicates the original index data on the evaluation system of organizational system development. $x_{tij}$ represents data standardized by the system.

#### The time-series value of cross-sectional data of organizational system development time point is calculated based on the *CRITIC* weight method.

In this paper, the cross-section data at the time point *t* in the development of organizational systems is used as the object of evaluation. Based on the above formula, the time-series value evolution of the cross-section data at the time point of organizational system development is defined as the sample data, and the time-series value of cross-sectional data at different time points in the process of organizational change and development is obtained by using the *CRITIC* weight method.

The *CRITIC* weight method makes use of the cross-sectional data of subsystem *i* at each time point, considers the contrast intensity and conflict between time points, and makes use of the objective attributes of the data itself to objectively weight the time-series value of the cross-sectional data of the time point in the process of organizational system development.

Form the original *CRITIC* weighted Index Data Matrix:


$$Z(x_{i})=\left(\begin{array}{ccc} Z(x_{11}) & \ldots & Z(x_{\partial 1})\\ \vdots & \ddots & \vdots \\ Z(x_{1n}) & \cdots & T_{\partial n} \end{array}\right)$$ (9)

The matrix $Z(x_{i})$ represents the value-benefit Set of time-value evolution of cross-sectional data at various time points in the transformation and development of organizational systems.

(1) Dimensionless processing

$$Z\left(x_{ti}^{'}\right)=\frac{Z(x_{ti})-Z{\left(x_{ti}\right)}_{\min}}{Z{\left(x_{ti}\right)}_{\max}-Z{\left(x_{ti}\right)}_{\min}}(\text{Positive indicators})$$ (10)

$$Z\left(x_{ti}^{'}\right)=\frac{Z{\left(x_{ti}\right)}_{\max}-Z(x_{ti})}{Z{\left(x_{ti}\right)}_{\max}-Z{\left(x_{ti}\right)}_{\min}}\left(\text{inverse indicators}\right)$$ (11)

(2) Time point differentiation

$$\begin{array}{c} S_{t}^{i}=\sqrt{\frac{\sum_{t=1}^{\partial }{\left(Z(x_{ti})-\bar{Z}(x_{ti})\right)}^{2}}{\partial -1}}\\ \bar{Z}(x_{ti})=\frac{1}{\partial }\sum_{t=1}^{\partial }Z(x_{ti}) \end{array}$$ (12)

The standard deviation represents the fluctuations in the time-value evolution benefits of the cross-sectional data of subsystem *i* at different time points within the time series. A larger standard deviation indicates greater variability in benefits at that time point, allowing for the release of more information.

(3) Conflict between time points

In terms of the correlation coefficient:


$$R_{t}^{i}=\sum_{t=1}^{\partial }\left(1-\left|r_{ti}\right|\right)$$ (13)

The correlation coefficient is used to express the correlation of each time point. The stronger the correlation between the time point cross-section data, the less conflict between the two, and the more the same information, the more the evaluation content will be repeated. To a certain extent, it weakens the evaluation strength of the index. The cross-sectional data of the organization’s system development has less time-series value weight at that time point.

(4) The amount of information

$$C_{t}^{i}=S_{t}^{i}*\sum_{j=1}^{p}\left(1-\left|r_{ti}\right|\right)=S_{t}^{i}*R_{t}^{i}$$ (14)

The more significant the amount of information, the greater the time-series value *t* of the cross-sectional data of the time point in the whole time series, and the more weight should be assigned.

(5) The time-series value weight of the cross-sectional data of the organization system at the time point *t*:

$$Q_{t}^{i}=\frac{C_{t}^{i}}{\sum_{t=1}^{\partial }C_{t}^{i}}$$ (15)

### Identification method of the order parameter of the organizational system considering the time-series value

The application of the *CRITIC* method ensures that the model can flexibly adjust the relative weights of the order parameters according to the time value variations across different periods. This adjustment directly influences the calculation of the order parameters for both the system as a whole and individual subsystems.

(1) The calculation of the population order parameter considering the time-series value

The dominant order parameter of the system at each time point calculated by the above formulas, combined with the time-series value weight coefficient calculated by the *CRITIC* method, is synthesized from the population. In this paper, a group dominant order parameter ${\overset{⌢}{\lambda }}_{ij}$ of the system considering time-series value is obtained.


$${\overset{⌢}{\lambda }}_{ij}=\sum_{t=1}^{\partial }Q_{t}^{i}\lambda_{tij}$$ (16)

$$t=(1,2,...,\partial );j=\left(1,2,...,p\right);i\text{=}\left(\text{1,2,}...\text{,n}\right)$$ (17)

(2) The calculation of individual the order parameter considering the time-series value

By integrating the time-value weight coefficients calculated using the *CRITIC* method, a comprehensive value parameter structure ${W^{\prime }}_{i}$ is derived for the evaluation objects, reflecting their individual value structures at each time point while considering the time value. Then the improved order parameter calculation method is used for the dominant order parameter ${\lambda^{\prime }}_{ij}$.

The comprehensive value parameter structure of time-series value is considered:


$${W^{\prime }}_{i}=\sum_{t=1}^{\partial }Q_{i}^{t}W_{ij}^{t}$$ (18)

$$t=(1,2,...,\partial );j=\left(1,2,...,p\right);i\text{=}\left(\text{1,2,}...\text{,n}\right)$$

An ideal synthetic value parameter structure:


$${\tilde{W}}^{\text{*}}=\frac{1}{n}\overset{n}{\underset{i=1}{\sum }}{W^{\prime }}_{i}$$ (19)

The cluster analysis of the integrated value parameter structure ${W^{\prime }}_{i}$ by SPSS can obtain the integrated theme of each subsystem. The structural similarity coefficient of each theme and the ideal comprehensive value parameter ${\tilde{W}}_{R}^{\text{*}}$ is calculated respectively, and the similarity degree is the highest. The theme is determined as the dominant order parameter.

The structural similarity coefficient of the main melody and the ideal comprehensive value parameter:


$$d_{r}\text{=}\sqrt{\overset{i=1}{\underset{n}{\sum^{\text{​}}}}{\left({\tilde{W}}_{R}^{*}-{\tilde{W}}_{t}^{*}\right)}^{2}}$$ (20)

$${\text{ρ}}_{R,Y}=\frac{1}{1+{\text{d}}_{r}}$$ (21)

## Control strategy of the identification result

### Control strategy of the result analysis of the order parameter identification under the time point *t*

Based on the original order parameter identification model, the time factor *t* is added to identify the dominant order parameter at each time point in the process of organizational system change and development. Taking the time-point *t* recognition results as an example, the determination of whether it can be the dominant order parameter is based on the similarity between the main melody and the ideal value parameter structure. The closer it is to the ideal value parameter structure ${\tilde{W}}^{\text{*}}$ the stronger the effect in the system is, and the higher the identification degree in the system group is. Therefore, the dominant order parameter at the time point *t* represents the comparative advantage of the organization system at that time point. It lays a foundation for the subsequent analysis of the changes of comparative advantage in the development of the social organization system.

### Based on the *CRITIC* weight method, the results of the time-series value calculation of organizational development are analyzed

As can be seen from the above, because of the diversification of factors affecting time-series value and the complexity of time-series value evaluation, the evaluation index system is constructed using the time-series value evolution benefit function $Z(x_{ti})$ of the cross-section data at each time point developed by the organization system, the time-series value benefit of the cross-section data at each time point of the system is evaluated. In this paper, the time-series value of the system at each time point is expressed intuitively and effectively by the weight coefficient $Q_{i}^{t}$ of the system at each time point. According to the time-series value weight, the contribution proportion of the system at a certain point in the process of achieving the evolutionary goal is analyzed.

### The analysis of the identification result of the order parameter of the organization system considering the time-series value

There are two aspects in order parameter identification considering time-series value: one is to synthesize the time-series value weight of the dominant order parameter from the system group, based on the macroscopic view, the dominant order parameter ${\overset{⌢}{\lambda }}_{ij}$ of the system group considering the time-series value is obtained, which directly represents the law of comparative advantage in the development process of the organization system considering the time-series value. Based on the analysis of the dominant order parameters of each time point, we can know the trend and law of comparative advantage change in the system development process. Secondly, from the microscopic point of view, the comprehensive value parameter ${\tilde{W}}^{\text{*}}$ corresponding to each individual and each object is calculated by using the dominant structure of each time point of the system. Then the dominant order parameter ${\overset{⌣}{\lambda }}_{ij}$ considering the time-series value of the system individual is identified. If the comprehensive ideal value parameter ${\tilde{W}}^{\text{*}}$ is taken as the benchmark, the dominant order parameter will keep its advantages and play a dominant role. Suppose the dominant order parameter could be better. In that case, the value parameter structure should be adjusted, following the example, and continue increasing investment to strengthen the dominant and dominant role. Corresponding to each individual put forward different regulatory strategies.

### Robustness test

To assess the robustness of the method proposed in this study, a sensitivity analysis will be conducted on the weights derived from the *CRITIC* method. The aim is to evaluate the impact of weight variations on the identification of order parameters and the overall conclusions of the study. By systematically varying the weights within a reasonable range and observing the changes in model outputs, the results of this sensitivity analysis will provide insights into the stability of the method and the importance of weight allocation. The main steps are as follows:

(1) Define the Range of Weight Variation: Determine a reasonable range for weight variation. Based on the original weights calculated by the *CRITIC* method, increase or decrease the weights by 10% and 20%, respectively, while ensuring that the sum of all weights remains 1 after each adjustment.
(2) Recompute Model Outputs: Under each scenario of weight variation, rerun the model to compute the corresponding results for the identification of order parameters.
(3) Analyze Differences in Results: Compare the results of the dominant order parameter identification under different weight scenarios and analyze the specific impact of weight changes on model outputs to assess the robustness of the study’s conclusions.

## Case study

### Raw data calculation

The level of high-quality development of the manufacturing industry in 29 provinces and cities in China during the five-year period of 2016–2020 was calculated using the proposed formula. Data from the China Statistical Yearbook of Industrial Economy, China Statistical Yearbook of Industry, China Statistical Yearbook of High-Tech Industries, China Statistical Yearbook of Science and Technology, China Statistical Yearbook, China Information Yearbook, China Statistical Yearbook of the Environment, and the China Energy Statistics Yearbook were utilized. The regulated industry replaces the missing data.

**Fig 1:**
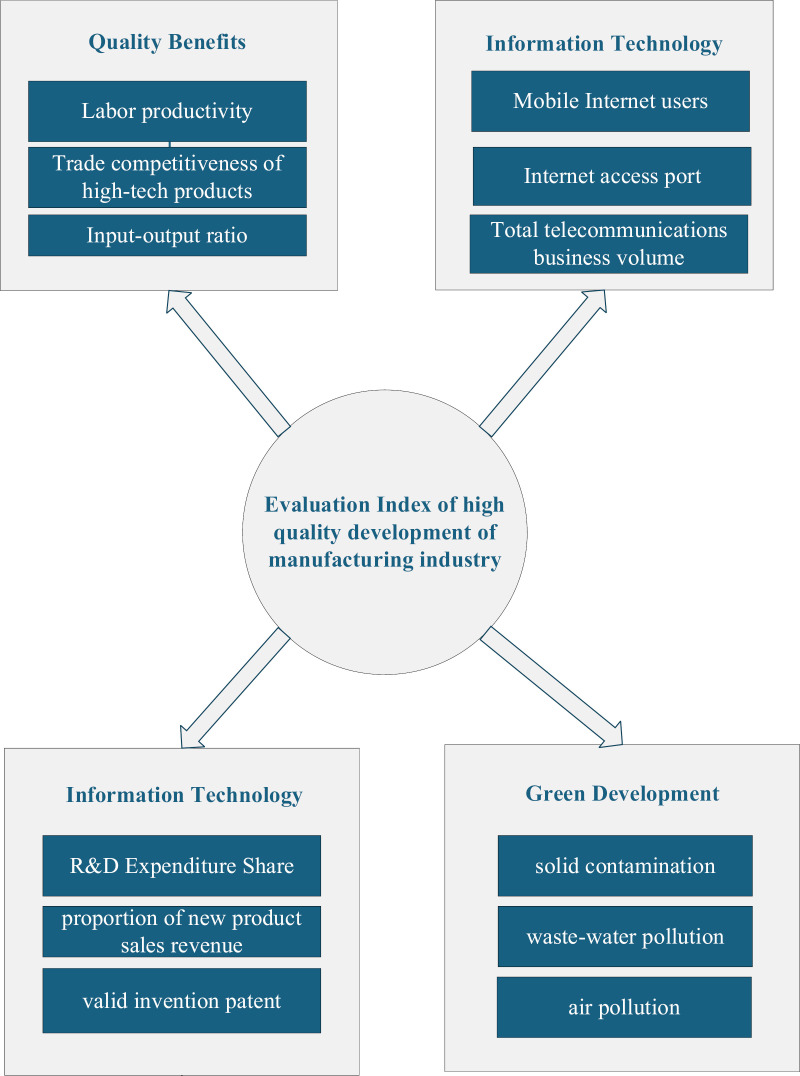
The Evaluation Index of high-quality development of the manufacturing industry.

A system for evaluating the high-quality development of China’s manufacturing industry has been constructed. This system evaluates the industry’s quality and efficiency, innovation capability, information technology, and green development, as shown in Fig 1.

To facilitate the calculation, the index system is numbered 29 provinces, of which 29 provinces are numbered 1–29 respectively, and the index system is shown in Table 1.

**Table 1:** Data acquisition and processing.

Numbering	Index criteria layer	Numbering	Index scheme layer
A	Quality benefits	A1	Labor productivity
		A2	Trade competitiveness of high-tech products
		A3	Input-output ratio
B	Capacity for innovation	B1	R&D Expenditure Share
		B2	The proportion of new product sales revenue
		B3	R&D personnel
		B4	Valid invention patent
C	Information technology	C1	Mobile Internet users
		C2	Internet access port
		C3	Total telecommunications business volume
D	Green development	D1	Solid contamination
		D2	Wastewater pollution
		D3	Air pollution

### Calculation of high-quality development order parameter of Chinese manufacturing industry considering the time-series value

#### Identification of the order parameter at each time point of high-quality development of China’s manufacturing industry.

Based on the above formula, the primary data of the indicator system are calculated to obtain the dominant order parameter of the high-quality development of China’s manufacturing industry every year from 2016 to 2020, as shown in Table 2:

**Table 2:** The dominant order parameter of high-quality development of China’s manufacturing industry at each time point.

Time point	The dominant order parameter												
2016	0.2491	0.1311	0.0309	0.0173	0.0185	0.0179	0.0161	0.0232	0.0914	0.0206	0.1952	0.0242	0.1689
2017	0.3129	0.0492	0.0196	0.0153	0.0164	0.0165	0.0141	0.0411	0.1240	0.0204	0.1933	0.0245	0.1526
2018	0.0072	0.0657	0.0073	0.0321	0.0321	0.0340	0.0299	0.0462	0.3431	0.0369	0.1014	0.0441	0.2201
2019	0.1262	0.0580	0.0742	0.0077	0.0071	0.0072	0.0060	0.0183	0.1167	0.0132	0.3798	0.0170	0.1686
2020	0.0085	0.0938	0.0074	0.0100	0.0080	0.0102	0.0057	0.0272	0.2589	0.0214	0.1636	0.0323	0.3530

#### Based on the *CRITIC* weight method, the time-series value of high-quality development of China’s manufacturing industry is calculated.

To evaluate the level of high-quality development of the manufacturing industry more accurately and put forward more targeted suggestions, it is necessary to establish the time-value evolution benefit function of cross-section data in each year based on the constructed evaluation indexes, to explore the annual time-series value evolution benefit of the high-quality development of China’s manufacturing industry from 2016 to 2020. The calculation of 9 based on the formula is shown in Table 3.

**Table 3:** 2016–2020 time-series value evolution benefit of high-quality development of China’s manufacturing industry.

	2016	2017	2018	2019	2020
Beijing	1191427	1321264	1644106	1063342	1110504
Tianjin	1307334	1660439	1730442	1892249	1662589
Hebei	747632.9	684256.2	807411.1	920580.6	944614.7
Shanxi	1009321	1397019	1506029	1633919	1682617
Neimenggu	646690.7	987928.5	1350380	1337573	1845229
Liaoning	994665.5	1249285	1511229	1729676	1714844
Jilin	896660.9	1266196	1537965	1836623	1815347
Heilongjiang	872426.2	883988.1	1028547	1663496	1713932
Shanghai	925164.5	1388785	1691848	1921059	2048994
Jiangsu	4381327	5730643	3605550	3658014	3410079
Zhejiang	1124322	1384552	1799847	2251647	2357955
Anhui	1077096	1356113	1645720	1911032	1986441
Fujian	1177501	1533880	1771744	1999902	2152816
Jiangxi	1046040	1317255	1585603	1901131	2001395
Shandong	1259716	1546350	1491010	1614781	1412245
Henan	1427542	1082267	1569359	1891355	1847478
Hubei	1115475	1354710	1618945	1936719	2041676
Hunan	1187611	1449921	1678644	1861353	1870785
Guangdong	16311587	20436217	24681976	27801698	28594128
Guangxi	995397.4	1166146	1608409	1902326	1777076
Hainan	1720780	172310.2	8531629	341894.4	222275.3
Chongqing	1223891	1476368	1828586	1989552	1971538
Sichuan	920946.6	723082.6	1365028	1399248	1309633
Guizhou	1012654	1281460	1603640	1843831	1781294
Yunnan	1018039	979679.3	1478116	1693836	975861.6
Shaanxi	702304.8	1021992	1529855	1754900	1733721
Gansu	970048.5	1315961	1802506	2236018	1926668
Qinghai	33703.17	1024858	142023.9	7675934	2133638
Ningxia	1033882	1087565	1847067	1962090	1349688

According to Formula 15, the calculation matrix is made up of the time-series value evolution benefit of the high-quality development cross-section data of every province and city every year, The time series value weights of high-quality development of China's manufacturing industry from 2016 to 2020 are calculated, as shown in Table 4.

**Table 4:** 2016–2020 weight coefficient of the time-series value of high-quality development of China’s manufacturing industry.

Time point	Weight of time-series value
2020	0.1380
2019	0.2497
2018	0.3143
2017	0.1543
2016	0.1437

#### Calculation of high-quality development order parameter of Chinese manufacturing industry group considering the time-series value.

(1) The dominant order parameter calculation of high-quality development of China’s manufacturing industry group considering the time-series value

According to Formula 16, as well as Tables 1 and 3, the time-series value weighting coefficients of the dominant order parameter and the annual cross-sectional data of the high-quality development of China’s manufacturing industry at each time point are calculated respectively, from 2016 to 2020, the dominant order parameter ${\overset{⌢}{\lambda }}_{ij}$ driving the overall high-quality development of China’s manufacturing industry is obtained. ${\overset{⌢}{\lambda }}_{ij}$ (0.1190 0.0745 0.0293 0.0182 0.0182 0.0190 0.0162 0.0325 0.2050 0.0239 0.2072 0.0298 0.2078).

(2) Calculating the dominant order parameter of high-quality development of China’s manufacturing provinces considering the time-series value

The individual value structure of each province from 2016 to 2020 was synthesized, and the comprehensive value parameter structure considering the time-series value of each province was obtained, as shown in Table 5.

**Table 5:** Structure of integrated value parameters for each province.

	A1	A2	A3	B1	B2	B3	B4	C1	C2	C3	D1	D2	D3
Beijing	0.2355	0.0057	0.6831	0.0073	0.0069	0.0069	0.0069	0.0103	0.0094	0.0089	0.0057	0.0075	0.0059
Tianjin	0.6911	0.1131	0.1151	0.0083	0.0087	0.0086	0.0072	0.008	0.0087	0.0076	0.0071	0.0088	0.0077
Hebei	0.0065	0.0394	0.0054	0.0042	0.0041	0.0043	0.004	0.0104	0.0166	0.0073	0.4707	0.0078	0.4193
Shanxi	0.019	0.0785	0.0097	0.0067	0.0067	0.0068	0.0067	0.0099	0.0112	0.0088	0.7977	0.009	0.0293
Neimenggu	0.3227	0.0128	0.0065	0.0053	0.0053	0.0053	0.0053	0.0073	0.0075	0.0067	0.5203	0.0066	0.0884
Liaoning	0.0243	0.0232	0.0113	0.0073	0.0071	0.0074	0.0071	0.0122	0.0216	0.0106	0.8266	0.012	0.0291
Jilin	0.8038	0.0126	0.019	0.007	0.0069	0.0071	0.0068	0.0156	0.0291	0.0115	0.0166	0.0134	0.0506
Heilongjiang	0.4942	0.0174	0.0168	0.0056	0.0054	0.0059	0.0054	0.016	0.0418	0.0102	0.0193	0.0125	0.3495
Shanghai	0.7749	0.0448	0.0705	0.0105	0.0081	0.0095	0.0085	0.0141	0.0158	0.0123	0.0074	0.0147	0.0091
Jiangsu	0.0077	0.0913	0.0084	0.0168	0.0220	0.0228	0.0083	0.0382	0.6558	0.0311	0.0108	0.0478	0.0389
Zhejiang	0.0085	0.6842	0.024	0.0101	0.0091	0.013	0.0073	0.0227	0.1614	0.0226	0.0077	0.0177	0.0116
Anhui	0.0117	0.8022	0.0157	0.0083	0.0082	0.0086	0.0075	0.0191	0.0295	0.0136	0.0232	0.017	0.0355
Fujian	0.007	0.8461	0.0200	0.0100	0.0088	0.0101	0.0078	0.0137	0.0265	0.0138	0.0098	0.0148	0.0115
Jiangxi	0.0087	0.8753	0.0092	0.008	0.008	0.0082	0.0075	0.0110	0.0135	0.0103	0.0141	0.0121	0.0142
Shandong	0.0098	0.0423	0.0093	0.0089	0.0076	0.0087	0.0063	0.0325	0.1551	0.0157	0.0307	0.0314	0.6419
Henan	0.0063	0.4948	0.0086	0.0063	0.0082	0.0068	0.0056	0.037	0.1427	0.0202	0.0224	0.0254	0.2159
Hubei	0.011	0.8478	0.0100	0.0094	0.0082	0.0090	0.0079	0.0154	0.0221	0.0129	0.0126	0.0183	0.0155
Hunan	0.0075	0.8482	0.0114	0.0084	0.0083	0.009	0.0074	0.0183	0.0192	0.0140	0.0120	0.0182	0.0179
Guangdong	0.0011	0.009	0.0032	0.1233	0.1233	0.1233	0.1233	0.1233	0.1175	0.1233	0.0014	0.1233	0.0045
Guangxi	0.0104	0.8216	0.0242	0.0069	0.0069	0.007	0.0069	0.0196	0.0294	0.0144	0.0151	0.0171	0.0205
Hainan	0.8967	0.0163	0.0108	0.0075	0.0074	0.0075	0.0075	0.0079	0.0082	0.0078	0.0074	0.0077	0.0074
Chongqing	0.0111	0.8800	0.0112	0.0082	0.0089	0.0082	0.0075	0.0115	0.0142	0.0105	0.0085	0.0103	0.0099
Sichuan	0.0102	0.4131	0.0099	0.0067	0.006	0.0068	0.0058	0.0658	0.4023	0.018	0.0177	0.0175	0.0202
Guizhou	0.0213	0.8638	0.0218	0.0074	0.0073	0.0075	0.0073	0.0107	0.0100	0.0113	0.0109	0.0088	0.0120
Yunnan	0.3038	0.5179	0.0362	0.0057	0.0057	0.0058	0.0057	0.0150	0.0133	0.0194	0.0456	0.0108	0.0153
Shaanxi	0.0899	0.7622	0.0161	0.0081	0.0068	0.0083	0.0068	0.0151	0.0181	0.0134	0.0158	0.0106	0.0287
Gansu	0.7602	0.1415	0.0096	0.0071	0.0071	0.0071	0.0071	0.0099	0.0100	0.0093	0.0109	0.0082	0.0119
Qinghai	0.7097	0.2154	0.006	0.0059	0.0059	0.0059	0.0059	0.0059	0.0059	0.0059	0.0158	0.0059	0.0061
Ningxia	0.1963	0.7210	0.0156	0.0064	0.0064	0.0064	0.0063	0.0065	0.0065	0.0065	0.0075	0.0065	0.0082

The ideal composite value parameter structure ${\tilde{W}}^{\text{*}}$ considering time-series value is calculated as follows: (0.2228 0.3876 0.0420 0.0118 0.0117 0.0121 0.0108 0.0208 0.0697 0.0165 0.1025 0.0180 0.0737).

By using SPSS to cluster and analyze the comprehensive value parameter structure of each province and city considering time-series value, the sweeping main melody ${\tilde{W}}_{R}^{\text{*}}$ of 29 provinces and cities belonging to different groups is calculated, based on Formula 20, the similarity coefficients between the main themes and the composite value parameter structure considering time-series value are calculated, as shown in Table 6.

**Table 6:** Province and city and their value parameter structure under each theme of high-quality development of the manufacturing industry.

Provincial and municipal groups	7 9 21 27 28 2 8 1 4 6 5													Similarity coefficient
Theme 1	0.5211	0.0619	0.0871	0.0071	0.0069	0.0071	0.0067	0.0106	0.0154	0.0091	0.2032	0.0097	0.0541	0.6853
Provincial and municipal groups	8 1 4 6 5													
Theme 2	0.2191	0.0275	0.1455	0.0065	0.0063	0.0065	0.0063	0.0112	0.0183	0.0091	0.4339	0.0095	0.1004	0.6649
Provincial and municipal groups	4 6 5													
Theme 3	0.1220	0.0382	0.0092	0.0065	0.0064	0.0065	0.0064	0.0098	0.0134	0.0087	0.7149	0.0092	0.0489	0.5800

According to the similarity between theme one and the ideal value parameter, it is concluded that theme one is the dominant order parameter of high-quality development of China’s manufacturing provinces considering time-series value. as follows ${\tilde{W}}_{R}^{\text{*}}$ (0.5211 0.0619 0.0871 0.0071 0.0069 0.0071 0.0067 0.0106 0.0154 0.0091 0.2032 0.0097 0.0541).

### Analysis of identification results

#### Analysis of the change and law of comparative advantage of high-quality development of China’s manufacturing industry considering the time-series value.

(1) Order parameter analysis at different time points: the comparative advantage of high-quality development of China’s manufacturing industry in 2016–2020:

They are manufacturing quality benefits in the early years of the development of advantages year by year, in 2018, to reach the lowest point. In innovation and information technology, the manufacturing industry in the early development of the advantages of the general upward trend, the most significant advantage in 2018, the latter advantage is no longer strong. The advantage of the manufacturing industry in the later stage of green development is especially remarkable.

(2) Analysis of comparative advantage considering time-series value: analysis of the law of comparative advantage of high-quality development of China’s manufacturing industry considering the time-series value

**Fig 2:**
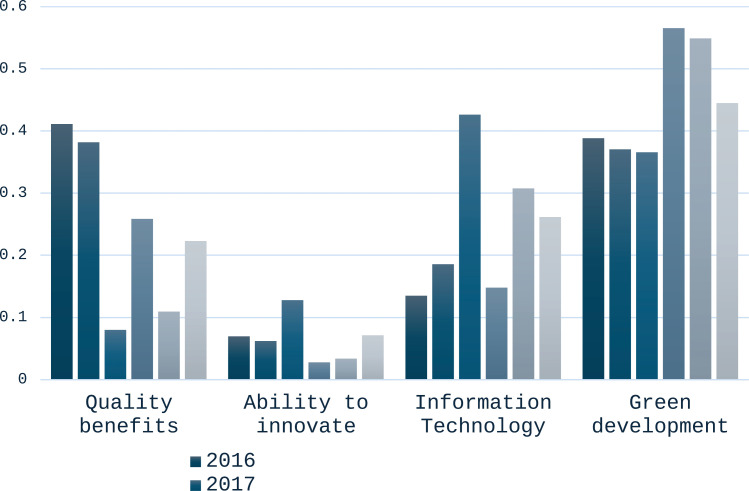
Manufacturing comparative advantage analysis for 2016–2020 considering the time-series value.

The advantage of high-quality development of the manufacturing industry shows its strong advantage in green development and information technology, and its development level is excellent in quality benefit, but it lacks innovation ability. According to the law of development, China’s manufacturing industry has made remarkable achievements in green development to realize high-quality development in recent years. There is a need to maintain existing development strengths and invest more in areas of weakness, such as innovation capacity. As shown in Fig 2.

#### The change law of time-series value of high-quality development of China’s manufacturing industry.

**Fig 3:**
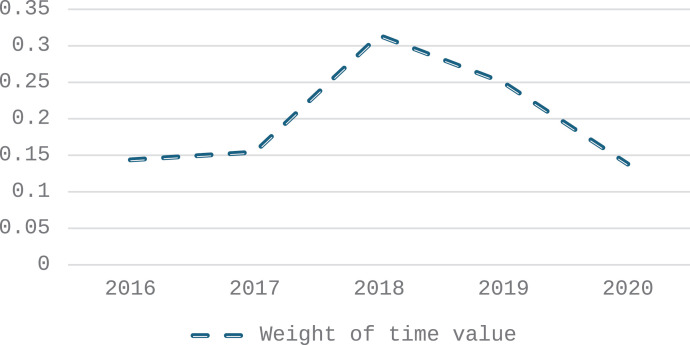
The time-value change trend of manufacturing high-quality development from 2016 to 2020.

China’s manufacturing industry under the general environment mainly presents in the early stage of the epidemic. The high-quality development of China’s manufacturing industry is in the stage of rapid growth, especially in 2018. The achievements of China’s manufacturing industry have laid a solid foundation for the high-quality development of the follow-up manufacturing industry. After the outbreak of the epidemic, China’s manufacturing industry, although the level of high-quality development is significant compared with 2018, still presents a late high-quality development value decline trend. Combined with Fig 3, the comparative advantage of the quality benefit of China’s manufacturing industry has declined after the epidemic, indicating that the development level of high quality in China’s manufacturing industry has fluctuated.

#### Recommendations for regulating high-quality development of manufacturing industry considering time-series values.

Drawing upon the theory of advantage perspective and the principle of advantage complementarity, each social organizational system inherently possesses unique capabilities and resource endowments, thereby engendering comparative advantages and complementarity. In suitable contexts, harnessing the intrinsic strengths of social organizational systems to maximize their efficacy becomes imperative. Consequently, this study proffers regulatory propositions aimed at both the holistic advancement and high-quality progression within diverse regional realms of China’s manufacturing industry.

(1) Regulatory recommendations for the holistic high-quality development of China’s manufacturing industry considering time-series values

**Fig 4:**
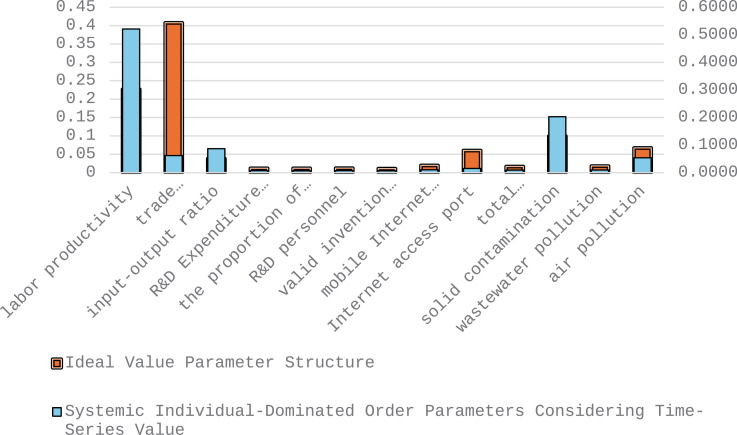
Dominant order parameters for high-quality development in manufacturing industries across provinces considering time-series value.

As delineated in Fig 4, the endeavor is to judiciously leverage the merits of production factors and internal reservoirs to optimize overarching advantages. Through a comparative analysis juxtaposing the dominant order parameters governing high-quality developmental trajectories across manufacturing sectors in different provinces against the envisaged ideal value parameter structure, the following strategic adjustments are advocated: judiciously curtail investments in labor productivity, input-output ratios, and solid waste management, while intensifying focus on enhancing competitiveness in high-tech product trade, internet accessibility nodes, and wastewater treatment standards. Accentuating the preeminence of order parameters in fostering high-quality developmental trajectories spanning provincial demarcations within the manufacturing domain emerges as quintessential to realize equitably balanced and high-caliber industry-wide advancement.

(2) Recommendations for regulating regional high-quality development in China’s manufacturing industry considering time-series values

**Fig 5:**
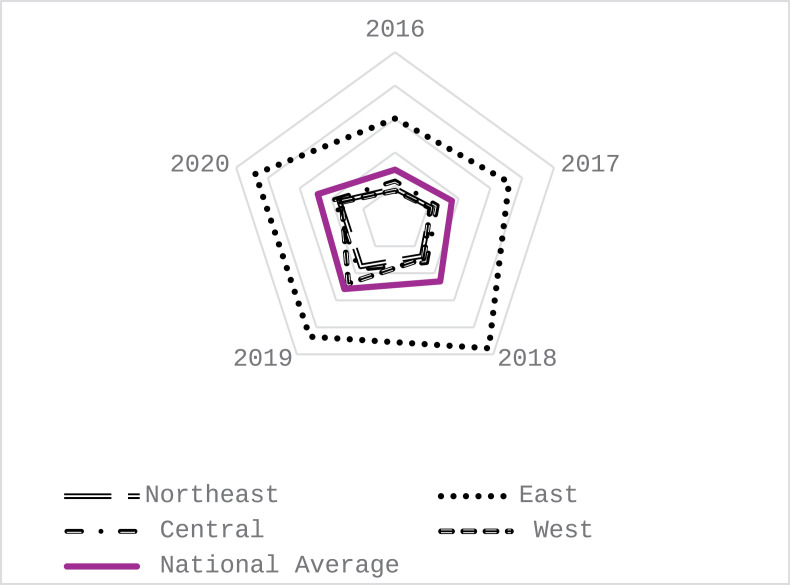
Evolution of time-value benefits in high-quality development of manufacturing industry across regions from 2016 to 2020.

From Fig 5, it is evident that during the period from 2016 to 2020, the eastern region of China exhibited a significant lead in high-quality manufacturing development compared to other regions nationwide. Concurrently, the central region experienced a steady ascent in its high-quality manufacturing landscape. However, the developmental pace in other regions trailed behind the national average. Despite not holding a dominant position, the northeastern region highlighted notable progress in high-quality manufacturing development, albeit with a noticeable deceleration in growth in 2020. In contrast, the western region’s high-quality manufacturing development remained relatively subdued on a national scale, although it managed to outpace the central and northeastern regions in 2018–2019. While the short-term impacts of the pandemic may temporarily dampen high-quality manufacturing outputs, its longer-term effects on the industry are anticipated to be limited, particularly with the eastern, central, and northeastern regions already demonstrating promising upward trends. This underscores the imperative of fostering regional synergy in high-quality manufacturing development as a pivotal component of the evolving economic landscape. Consequently, concerted efforts should be directed towards leveraging the eastern region's leadership, sustaining the developmental momentum of the central region, and bolstering resource allocation to underperforming regions to realize a regional economic framework characterized by complementarity and high-caliber development.

### Model robustness test analysis

(1) Assessing model sensitivity to weight changes

To evaluate the sensitivity of the model to variations in weights, this study specifically selected the year 2018 from the time series for weight adjustment. This selection was since the weight for 2018 is the highest (0.3143), implying a more significant impact on the model’s output. Consequently, adjusting the weight for 2018 can effectively reflect the model’s sensitivity during a critical period. Specifically, the weight for 2018 was increased and decreased by 10% and 20%, respectively, and the weights for other years were adjusted proportionally to ensure that the sum of all weights remained at 1. The adjusted time series weights are detailed in Tables 7 and 8.

**Table 7:** Time-series weights adjustments 10%.

Year	Original Weight	Weight + 10%	Weight −10%
2016	0.1437	0.1395	0.1480
2017	0.1543	0.1508	0.1588
2018	0.3143	0.3457	0.2829
2019	0.2497	0.2432	0.2562
2020	0.1380	0.1208	0.1442

**Table 8:** Time-series weights adjustments 20%.

Year	Original Weight	Weight + 20%	Weight −20%
2016	0.1437	0.1305	0.1568
2017	0.1543	0.1401	0.1684
2018	0.3143	0.3772	0.2514
2019	0.2497	0.2268	0.2726
2020	0.1380	0.1254	0.1506

(2) Dominant order parameters from a macro perspective

From a macro perspective, the dominant order parameters of the system integrate the performance of the entire system at different time points, providing a comprehensive view to assess the overall development trend of the system. Based on the adjusted time weight order, this study calculated the dominant order parameters of the system group. By comparing the dominant order parameters under different weight scenarios, these comparisons serve as a standard for robustness testing.

(3) Results of robustness testing

**Fig 6:**
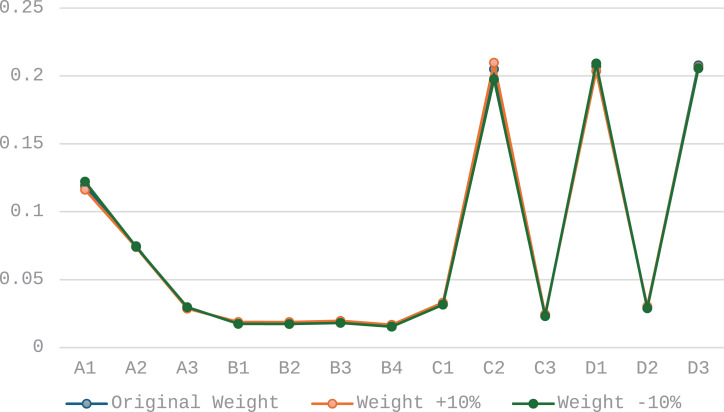
Dominant order parameter identification results with 10% time series weight adjustment.

**Fig 7:**
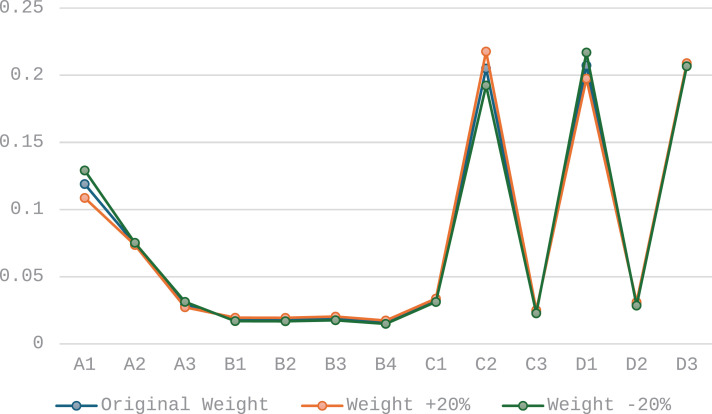
Dominant order parameter identification results with 20% time series weight adjustment.

The results presented in Figs 6 and 7 demonstrate that even with reasonable fluctuations of 10% and 20% in the weights, the identification results of the dominant order parameters remain essentially consistent. This finding strongly indicates the robustness of the proposed method against reasonable weight variations, thereby validating the reliability and applicability of the method.

## Discussion and conclusion

Extensive research explores the evolution of organizational systems, particularly within the manufacturing sector. However, much of this work remains constrained by the classical Haken model, which primarily addresses macro-level interactions of order parameters during system evolution. These studies tend to focus on static snapshots of system development, overlooking the time value embedded in cross-5 sectional data. As a result, the influence of temporal factors on organizational system evolution, especially in rapidly changing environments, remains underexplored.

This study addresses these limitations by incorporating time-series values into the identification of order parameters, offering a more dynamic and detailed framework for analyzing organizational systems. By integrating time-series data, this approach not only captures broad trends but also uncovers micro-level patterns of evolution, providing deeper insights into the internal and external factors driving organizational change. The proposed model, which combines group and individual order parameter identification with time-series analysis, enhances the precision of organizational performance evaluations and supports better decision-making in dynamic environments.

This research advances the field by presenting a time-series-based methodology for identifying order parameters, overcoming the key limitations of the classical Haken model. The combination of time-series analysis with the *CRITIC* method provides a more accurate reflection of system dynamics, showing how organizations evolve over time. This approach is particularly relevant to rapidly evolving manufacturing sector of China, where organizations must continuously adapt to shifting market conditions and strategic demands. Future research needs to explore the dynamic relationships between time points during organizational evolution, as these are critical to understanding the long-term development of complex systems.
